# Future orientation and perceived employability of chinese undergraduates: a moderated mediation model

**DOI:** 10.1007/s12144-022-03769-6

**Published:** 2022-10-13

**Authors:** Hao Chen, Yunhong Wu, Lin Jiang, Binfeng Xu, Xiaopei Gao, Wenjing Cai

**Affiliations:** 1 College of Economics & Management, Anhui Agricultural University, 230036 Hefei, China; 2 School of Public Affairs, University of Science and Technology of China, 230026 Hefei, China; 3 Institute of Intellectual Property, University of Science and Technology of China, 230026 Hefei, China; 4 Department of Management & Organisation, Vrije Universiteit Amsterdam, 1081HV Amsterdam, Netherlands

**Keywords:** Future orientation, Problem-based learning, Job market knowledge, Proactive personality, Perceived employability

## Abstract

Although scholars and practitioners have highlighted the significance of students’ attitudes for their future employment, few empirical examinations have attempted to determine the potential association between students’ future orientation and their perceived employability. Thus, drawing on career construction theory, we test the positive effect of students’ future orientation on their perceived employability by exploring the mediator of problem-based learning and the moderators of job market knowledge and proactive personality. Collecting our data via a time-lagged design (N = 368), we have found that the positive association between future orientation and employability is mediated by problem-based learning. Our moderation analyses further revealed that job market knowledge positively moderates the relationship between future orientation and problem-based learning and that students’ proactive personality positively moderates the relationship between problem-based learning and perceived employability.

## Introduction

Given the rapid development of the tourism industry, higher education on tourism has expanded rapidly in recent years (Barkathunnisha et al., 2019; Lugosi et al., 2017). Accordingly, the attraction of tourism management for undergraduate students has consistently garnered practitioners’ and researchers’ attention (Tolkach & Tung, 2019). Concerning such practice and research, a prominent topic is career management in terms of *employability* (Eurico et al., 2015). The main reason for highlighting this topic is that challenges in the tourism and hospitality industries reinforce students’ heavy employment pressure in their future job market (Reichenberger & Raymond, 2021), especially under the influence of the COVID-19 pandemic (Benaraba et al., 2022) Accordingly, today, the job prospects for those graduating majors in tourism and hospitality are far less promising than they used to be.

Consequently, the competitive environment of the employment process requires students to ask themselves the following question: “How can I make myself more employable as a graduating student to find a good job?” (Tymon, 2013). In response to this question, several studies have evaluated the factors that boost students’ perceived employability, i.e., “the individual’s perception of his or her possibilities of obtaining and maintaining employment” (Vanhercke et al., 2014). Researchers recognize the importance of personal factors, such as employability skills, for developing employability (Moreau & Leathwood, 2006; Tomlinson 2008). Future orientation is considered to be an important personal factor for successful career building. Scholars (Savickas & Porfeli, 2012; Wittekind et al., 2010) found that future orientation-related attributes, such as willingness to develop new competencies, opportunity awareness, positively influence perceived employability over time. Furthermore, Praskova & Johnston (2021) assessed the direct and indirect relationship between future orientation and career success (i.e. occupational fitness and employability) in the adult population with a sample of 285 adults. However, little empirical attention has been given to determining the potential association between students’ future orientation and their perceived employability (Tymon, 2013). To effectively foster students’ employability, scholars have suggested that the more advanced a student’s skills concerning his or her future orientation, i.e., an individual’s conscious and self-reported view of his or her future (Seginer & Rachel, 2008), the more adaptable to his or her employment environment the individual is. Therefore, examining the relationship between students’ future orientation and perceived employability is both timely and necessary to better understand the likelihood of individuals with adequate preparation for their work in the future to be employable in these uncertain economic times.

Accordingly, to address the above research limitation, we draw on career construction theory to explore the salient intervening mechanism by testing the mediator of *problem-based learning*, i.e., a learning model that develops critical thinking and problem solving skills (Cai, 2013; Hmelo-Silver, 2004). The basic assumption of career construction theory is that individuals can initiate an action-oriented process via certain behaviors regarding their career development (e.g., employability) (Savickas & Porfeli, 2012). Thus, we expect that students who have thoughts and feelings about their future, i.e., who envisage multiple possible future scenarios (Atance & O’Neill, 2001; Stoddard et al., 2011) tend to focus on specific behaviors, thereby addressing potential future problems, which in turn fosters their employability early in their careers.

According to career construction theory, scholars have also suggested that individuals’ personal characteristics (e.g., self-career management) partially facilitate their career development (Chan et al., 2015). We therefore propose job market knowledge and proactive personality as boundary conditions for the association between future orientation and employability via problem-based learning. Specifically, when students have more knowledge about their job market (e.g., job searching and required skills), they are particularly prone to transfer their subjective view of the future to specific issues (e.g., learning how to solve workplace-based problems in the future).

Career construction theory also suggests that proactive individuals are more likely to navigate increasingly complex and challenging career paths (Berg et al., 2010), which may facilitate their perception of being more employable. Proactive students are apt to take an action that influences and changes their undesirable surroundings (Hirschi et al., 2013). Therefore, during problem-based learning where students are involved in learning environments concerning job-related skills and knowledge (Liu et al., 2020), students are more proactive in overcoming difficulties in terms of learning how to handle problems, enabling them to be well-equipped with employable abilities.

**Fig. 1:**
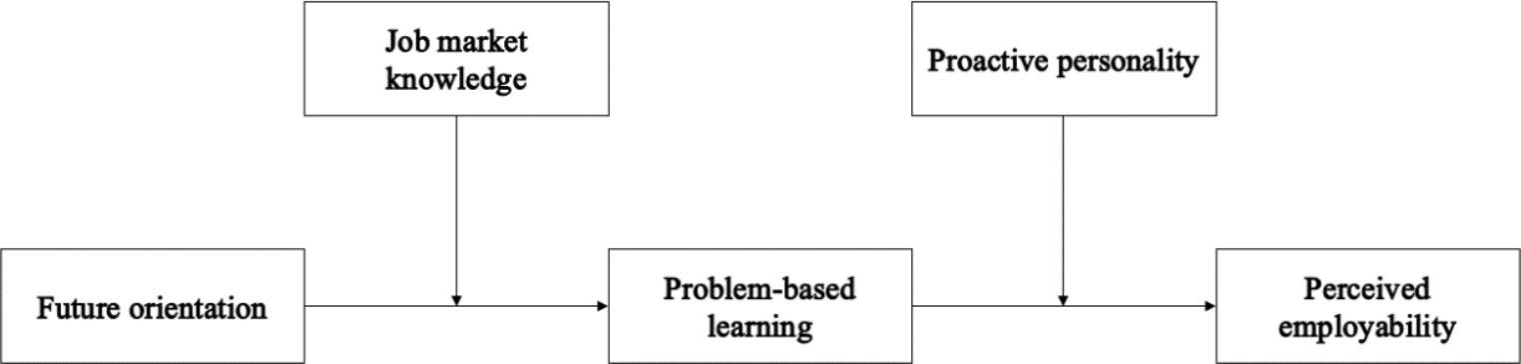
The hypothesized model

Accordingly, in this study, we empirically examine the relationship between students’ future orientation and their perceived employability by exploring the mediator of problem-based learning and the moderators of job market knowledge and proactive personality. Our hypothesized model is shown in Fig. 1.

By addressing the research question of *why, how and when does students’ future orientation influence their perceived employability*, the current study contributes to the current literature in three ways. First, we are among the first to identify the potential relationship between students’ future orientation and their perceived employability, thereby enriching the existing knowledge on the predictors of students’ employability. Second, our examination of the mediator of problem-based learning opens the black box of the influence of future orientation on employability from a behavioral perspective. Finally, by identifying the boundary conditions of job market knowledge and proactive personality, we extend the literature by accounting for personal-dependent variables to examine the unique relationship between future orientation and employability among students majoring in tourism and hospitality.

## Theoretical background and Hypotheses Development

### Future orientation and perceived employability

In the era of VUCA, volatility, uncertainty, complexity, and ambiguity make lifetime employment and job security no longer the norm (Hall & Heras, 2010; Liu et al., 2019), which brings many new challenges to students’ employment prospects. For students, they need to evaluate their own situation and perceived employment opportunities while building their career (Tomlinson et al., 2018). Therefore, employability has become an important part of students’ career (Fugate et al., 2021; Guilbert et al., 2016), scholars have begun to replace the traditional outcome of career success with employability (Lo Presti & Pluviano, 2016). For student job seekers, after earning a degree, they may aspire to some level of continued employment to build their career. Therefore, students’ perceived employability is defined as their perceived ability to obtain sustainable employment in accordance with their own qualification level (Rothwell et al., 2008). We believe that students’ employability is a dynamic and developmental concept with future-oriented and progressive characteristics. It includes not only students’ obvious abilities, such as the ability to find satisfactory jobs, but also their potential for future career success, such as the ability to be employed for a long time and develop a career. The formation of students’ employability is closely related to the formation of their critical and reflective thinking skills, problem-solving abilities, self-management skills and other related abilities during learning (Makkonen, 2017).

Future careers are hard to predict, but humans have developed the ability to think and propose many possible future scenarios (Ginevra et al., 2016; Strauss et al., 2012). Future orientation refers to an individual’s thoughts, plans, motivations, hopes and feelings about their future. The individual’s future orientation skills grow together with their independence, personal identity, and self-regulation rising. As students grow, they begin to pay more attention to their future career direction (Cabras & Mondo, 2018). It is important for students to have a future orientation when making decisions about their careers and employment opportunities (Chua et al., 2015).

Future orientation provides the grounds for setting goals, planning, exploring options, and making commitments that guide the person’s behavior and development processes, which in turn enhance students’ employability (Brown et al., 2019; Jackson & Tomlinson, 2020). According to career construction theory (Savickas et al., 2005), access to career resources is important when individuals are actively constructing their careers (Savickas & Porfeli, 2012). Career resources help individuals achieve subjective and objective career success (Kozan et al., 2019; Peng et al., 2018), such as employability (Sibunruang et al., 2016). Future orientation is a useful career resource for career success. For example, future orientation and thinking about the future can increase the initiative and flexibility of student behavior and advance the successful achievement of set goals (Schacter et al., 2008). Specifically, the higher the level of future orientation, the more interested in their own career prospects, the more actively engaged in career exploration, and the higher the level of perceived employability (Cheung et al., 2018). Therefore, we believe that the level of future orientation of students is a source of motivation for them to increase their employability in their careers. In summary, we propose the following hypothesis:

#### Hypothesis 1

*Future orientation is positively related to students’ perceived employability.*

### Problem-based learning as a mediator

Nowadays, in order to remain competitive, individuals need to become independent problem solvers. The acquisition of knowledge alone is not enough to ensure that students solve real-world problems, but also requires students to develop the ability to think independently and solve problems (Liu et al., 2020). Problem-based learning, a learning model that has received much attention in recent decades (Chang et al., 2012; Dunlap, 2005), has been widely adopted in different fields and educational settings to promote critical thinking in real learning situations and problem-solving skills (Yew & Goh, 2016). Problem-based learning enables students to try new problem-solving methods, acquire new ways of thinking in different problem-solving situations, and develop autonomous learning habits through practice and reflection (Cai, 2013; Hmelo-Silver, 2004). Problem-based learning is committed to training competent and skilled practitioners and promoting the long-term development of the knowledge and skills acquired during their studies (Strobel & Van Barneveld, 2009).

Future orientation enables behaviors such as planning, problem solving, and success for future-related issues and solution-oriented attitudes toward those issues (Nuttin et al., 1985). For students, future orientation gives them a “prevent it before it happens” mentality that will allow them to engage in such activities as problem-based learning. Specifically, future orientation motivates students to actively learn how to deal with complex, chaotic, uncertain, and unknown problems in future life, and more importantly, develops their ability to evaluate the feasibility of solutions (Cho et al., 2015).

Problem-based learning helps students develop skills needed for flexible employment, such as critical thinking, thinking, solving, and reflective skills, which in turn improve their employability (Liu et al., 2020). Specifically, Problem-based learning students are able to actively respond to changing environments and new challenges. They are good at thinking out of the fixed way, thinking from multiple perspectives, mastering learning experience through problem solving and enhancing knowledge accumulation, which will enhance students’ employability (Almulla, 2020; Peng et al., 2018; Li et al., 2020)also confirmed the above point of view. This study adopted structural equation model to analyze 553 undergraduates in Taiwan, and the results showed that problem-based learning had a significant positive impact on students’ employability. In addition, Clausen & Andersson (2019) found through a case study that problem-based learning helps students master learning experiences and form good thinking patterns, which in turn contributes to their employability.

Career construction model (Savickas, 2013; Savickas & Porfeli, 2012) show that individuals are willing (i.e., adaptive readiness) and able (i.e., adaptability resources) to show their attitudes and behaviors (i.e., adapting responses) to cope with the changing environment when faced with events that have a significant impact on their career, such as epidemic, so as to achieve positive results such as career success (i.e., adaptation results) (Rudolph et al., 2017; Savickas, 2020; Šverko & Babarović, 2019). Future orientation serves as an adaptive resource that enables students to proactively build their careers and effectively respond to the opportunities and challenges presented by the environment, so that they can better develop and maintain their employability (Bridgstock, 2009; Forrier & Sels, 2003; Savickas & Porfeli, 2012). Specifically, future orientation will make students pay more attention to their own future development trends (i.e. adaptive readiness), have the ability to independently plan and choose future career development paths (i.e. adaptability resources), and at the same time promote students to participate in problem-based learning, with critical thinking responding to changes and challenges in a career (i.e. adapting responses), which in turn improves student employability (i.e. adaptation results) (Savickas & Porfeli, 2012). Therefore, we believe that problem-based learning plays an intermediary role in the relationship between future orientation and students’ perceived employability.

#### Hypothesis 2

*Problem-based learning mediates the relationship between future orientation is positively related to students’ perceived employability.*

### The moderator of Job Market Knowledge

Although we argue that the distal relationship between students’ future orientation and their perceived employability is mediated by problem-based learning, we expect a moderating effect on this relationship following career construction theory, i.e., some career-related resources (e.g., personal factors) may act as a booster to strengthen the association between predictors and career outcomes. Among these personal-related resources, scholars have suggested that individuals’ knowledge and skills resources function as their career-oriented assets, facilitating their career development (Yoopetch et al., 2021) Thus, in this section, based on career construction theory, we explore how two important characteristics of students—job market knowledge and proactive personality—moderate the influence of students’ future orientation on their perceived employability via problem-based learning.

We posit a positive moderating effect of job market knowledge on the future orientation–problem-based learning association. Specifically, job market knowledge represents the extent to which students grasp current labor market trends and developments (Marciniak et al., 2021). This type of supportive resource signals that students are resourceful via their occupational expertise in a particular area; that is, they are ready to handle predictable duties and work roles (Stoeber et al., 2016). Thus, via more self-motivations, such students are triggered to perform specific actions related to career development. They therefore have more possibilities for transferring their career-related directions to behaviors that facilitate addressing career-related problems.

Accordingly, when students have certain knowledge concerning their desired future occupation, they are well equipped with knowledge regarding what they will encounter after graduation (Gati & Kulcsár, 2021). Hence, their future orientation will favor their behaviors for addressing problems in their future workplace when students learn about these problems during their education. In contrast, when students obtain less knowledge about their future jobs and current job market, they are ill prepared for their future employment. Here, they have fewer intentions of engaging in activities relating to developing their career. As such, students without a future orientation will be less motivated to learn about problems. Thus, we propose that the positive effect of students’ future orientation on their problem-based learning will be more salient when they have a higher level of job market knowledge.

#### Hypothesis 3

*Job market knowledge positively moderates the relationship between students’ future orientation and their problem-based learning; thus, this relationship is stronger when students’ job market knowledge is high rather than low.*

### The moderator of proactive personality

Applying career construction theory, we also identify the moderator of proactive personality in the relationship between problem-based learning and perceived employability. Scholars investigating proactive personality—a type of stable disposition—have found that individuals with different levels of proactive personality show great differences in their behaviors in the environment (Parker et al., 2006). Specifically, individuals with a strongly proactive personality are less constrained by the external forces of their environment; instead, they tend to take the initiative to promote change by targeting opportunities in advance and taking initiatives to achieve goal realization (Bindl & Parker, 2011). In the career literature, researchers have indicated that proactive personality can predict students’ desirable career-related attitudes and behaviors (Pan et al., 2018) because such a personality renders them prone to developing and managing their careers (Hon et al., 2022).

As discussed above, problem-based learning involves improving students’ interests in learning and career paths (Clausen & Andersson, 2019; Li et al., 2020). When students have a highly proactive personality, they are more likely to have a sufficiently stable disposition to develop the appropriate learning attitudes and higher-order thinking skills needed to face real-world challenges in their future employment, e.g., critical thinking and reflection skills (White et al., 2004), which increases their positive attitude concerning being employable. In contrast, students with a less proactive personality are less likely to develop the appropriate learning attitudes for their future jobs, as they have less motivation to think about such behavior in advance. Hence, they fail to utilize their problem-based learning skills to develop their employable skills. We therefore expect that the positive effect of students’ problem-based learning on their perceived employability will be more salient when students have a highly proactive personality.

#### Hypothesis 4

*Proactive personality positively moderates the relationship between students’ problem-based learning and their perceived employability; thus, this relationship is stronger when students have a higher level of proactive personality.*

## Methods

### Sample and procedures

We employed the survey design in the current study. As our study targets college students majoring in tourism and hospitality management, we invited Chinese undergraduates to participate. Our sample was from a university in the middle area of China that was randomly selected from a list of universities based on a research project on the new area of tourism management. Specifically, as we employed time-lagged research, one author randomly submitted our questionnaire to the same students at two different time points to collect data. These participants were informed that the questionnaire was anonymous and that their responses would be kept confidential.

At Time 1, one author submitted an online questionnaire to 396 students who were willing to participate, and they completed the questionnaire after they were introduced to the topic of this research. They were asked to rate their future orientation, job market knowledge, problem-based learning, and proactive personality. They also reported their demographic information (i.e., age, gender, and internship experience). A total of 380 responses were received at Time 1. After four weeks, at Time 2, the other set of questionnaires was submitted to the same students, who were then asked to rate their perception of their employability. Ultimately, 368 responses were received, for a response rate of 92.9%. Among these students, who majored in tourism and hospitality management, most were female (59%, *SD* = 0.60). Their average age was 21.08 years old (*SD* = 1.77), and most of them had no internship experience (69.6%, *SD* = 0.46).

### Measures

Since all the scales were originally from English versions, the translation–back translation procedure was employed to translate all the scales from English to Chinese (Brislin, 1980). On this basis, we invited students to conduct pre-survey, and made minor adjustments to the items of the scale according to students feedback and expert opinions. In the pre-survey, each scale showed good reliability and validity.

#### Future orientation

We used a scale with 13 items from Santilli et al. (2017) to measure future orientation. A sample item was “I like to daydream about what my future holds for me”. Respondents rated each of the items on a 5-point scale from 1 = it describes me not at all to 5 = it describes me very well. The Cronbach’s α was 0.91.

#### Job market knowledge

We used a scale with 3 items from Hirschi et al. (2018) to measure job market knowledge. A sample item was “I have a good knowledge of the job market”. Respondents rated each of the items on a 5-point scale from 1 = not true at all to 5 = completely true. The Cronbach’s α was 0.90.

#### Problem-based learning

The problem-based learning scale compiled by Liu et al. (2020) consists of 6 items, including two dimensions: knowledge sharing and problem solving. Representative items such as “I can organize and prepare for small group sessions” and “I can utilize relevant resource materials effectively”. Respondents rated each of the items on a 7-point scale from 1 = strongly disagree to 7 = strongly agree. The Cronbach’s α coefficients of the two dimensions were 0.79 and 0.85.

#### Proactive personality

We followed Parker et al. (2006) and used a 4-item scale to measure proactive personality. A sample item was “No matter what the odds, if I believe in something, I will make it happen”. Respondents rated each of the items on a 5-point scale from 1 = not true at all to 5 = very true. The Cronbach’s α was 0.82.

#### Students perceived employability

A 16-item scale from Rothwell et al. (2008) was used to measure students perceived employability. Sample item was “I achieve high grades in relation to my studies”. Respondents rated each of the items on a 5-point scale from 1 = strongly disagree 5 = strongly agree. The Cronbach’s α was 0.89.

#### Control variables

We controlled the following variables: gender (1 = male; 2 = female; 3 = non-binary / third gender; 4 = prefer not to say), age (in years) and internship experience (1 = yes; 2 = no).

### Analytical Strategy

Firstly, we used SPSS 26.0 and Amos 26.0 to establish validity. Next, to test mediation and moderation effects in the current study, we used separate hierarchical multiple regression analyses to test hypotheses with SPSS 26.0. Finally, to further clarify the mediation effect, we employed the PROCESS program developed by Hayes (Preacher et al., 2007) in SPSS using a bootstrap procedure with 5,000 samples to produce a confidence interval (CI) for the indirect effect.

## Results

### Validity analyses

Before testing our hypotheses, we conducted a series of analyses to establish validity. First, due to the one-source data set (i.e., from employee ratings), we test for the presence of common method bias (CMB). Specifically, we performed Harman’s single factor test with principal axis factoring (PAF) as an extraction method to examine whether most of the variance could be explained by a single factor (Harman, 1976; Podsakoff et al., 2003). The results revealed multiple distinct factors, with the first unrotated factor accounting for only 26.24% of the total variance extracted. Thus, CMB was not a serious concern in our data. Second, we carried out Kaiser–Mayer–Orkin (KMO) test and Bartley spherical test (*p*-value) in SPSS before conducting CFA. The factor loading should be 0.50 or greater. The KMO value was 0.90 and Bartley spherical test (*χ2* [*df* = 861) = 8219.26, *p* < 0.001) revealed statistical significance, which indicates that this measurement model had good structural validity. Further, we conducted a confirmatory factor analysis (CFA) to check the measures’ discriminant validity of future orientation, job market knowledge, problem-based learning, proactive personality and perceived employability. As shown in Table 1, alternative models indicated a poor fit to the date—i.e., the four-factor model (χ^2^ /*df* = 3110.41/813.00, *p* < 0.001, SRMR = 0.09, RMSEA = 0.09, IFI = 0.70, TLI = 0.68, CFI = 0.70), the three-factor model (*χ*^*2*^ /*df* = 3807.17/816.00, *p* < 0.001, SRMR = 0.10, RMSEA = 0.10, IFI = 0.61, TLI = 0.59, CFI = 0.61), the two-factor model (χ^2^ /*df* = 4098.81/818.00, *p* < 0.001, SRMR = 0.10, RMSEA = 0.11, IFI = 0.58, TLI = 0.55, CFI = 0.57), and the one-factor model (χ^2^ /*df* = 5127.52/819.00, *p* < 0.001, SRMR = 0.12, RMSEA = 0.12, IFI = 0.44, TLI = 0.41, CFI = 0.44). Our proposed five-factor model provided a better fit to the data (χ^2^ /*df* = 1520.41/788.00, *p* < 0.001; Tucker-Lewis index (TLI) = 0.90, comparative fit index (CFI) = 0.91, incremental fit index (IFI) = 0.91, root mean square error of approximation (RMSEA) = 0.05, standardized root mean squared residual (SRMR) = 0.07) than all the alternative models. Values at or above 0.90 for the CFI, TLI and IFI, values at or below 0.08 for the RMSEA and values at or below 0.08 for the SRMR are considered indicates good fit (Hu & Bentler, 1999; Schumacker et al., 1996). Therefore, the results of the CFA revealed that all the goodness of-fit indexes were satisfactory. Taken together, the results provided strong support for the distinctiveness of the five study variables for subsequent analyses.

**Table 1:** Confirmatory factor analysis

CFA models	*χ* ^*2*^	*df*	SRMR	RMSEA	IFI	TLI	CFI
Five-factor model	1520.41	788.00	0.07	0.05	0.91	0.90	0.91
Four-factor model	3110.41	813.00	0.09	0.09	0.70	0.68	0.70
Three-factor model	3807.17	816.00	0.10	0.10	0.61	0.59	0.61
Two-factor model	4098.81	818.00	0.10	0.11	0.58	0.55	0.57
One-factor model	5127.52	819.00	0.12	0.12	0.44	0.41	0.44

* N* = 368; Five-factor model: Future orientation, Job market knowledge, Problem-based learning, Proactive personality, Perceived employability; Four-factor model: Future orientation + Job market knowledge, Problem-based learning, Proactive personality, Perceived employability; Three-factor model: Future orientation + Job market knowledge + Problem-based learning, Proactive personality, Perceived employability; Two-factor model: Future orientation + Job market knowledge + Problem-based learning + Proactive personality, Perceived employability; One-factor model: Future orientation + Job market knowledge + Problem-based learning + Proactive personality + Perceived employability

Table 2 presents the descriptive statistics, reliabilities and correlations of all the variables in the current study. Consistent with our expectations, future orientation is positively correlated with problem-based learning (*r* = 0.31, *p* < 0.01) and perceived employability (*r* = 0.37, *p* < 0.01). Problem-based learning is positively correlated with perceived employability (*r* = 0.35, *p* < 0.01). Finally, job market knowledge is positively related to problem-based learning (*r* = 0.29, *p* < 0.01) and proactive personality is positively related to perceived employability (*r* = 0.35, *p* < 0.01).

**Table 2:** Mean, standard deviations and correlations among the variables

	Mean	*SD*	1	2	3	4	5	6	7
1. Future orientation	3.75	0.69							
2. Problem-based learning	5.46	0.93	0.31^**^						
3. Job market knowledge	2.96	0.98	0.35^**^	0.29^**^					
4. Proactive personality	3.63	0.78	0.49^**^	0.23^**^	0.47^**^				
5. Perceived employability	3.50	0.63	0.37^**^	0.35^**^	0.37^**^	0.35^**^			
6. Gender	1.67	0.60	0.11^*^	0.07	-0.03	0.03	-0.13^*^		
7. Age	21.08	1.77	0.04	0.09	0.18^**^	0.09	0.07	0.03	
8. Internship experience	1.30	0.46	0.04	0.10	0.07	0.06	-0.02	0.04	0.02

*N* = 368; * *p* < 0.05, ** *p* < 0.01

### Hypotheses testing

We used hierarchical regression analyses to test mediation effects. In Table 3, the result indicates that after controlling for the effect of employees’ gender, age and internship experience, future orientation is positively associated with perceived employability (*β* = 0.38, *p* < 0.001), thus supporting H1. In addition, future orientation has a positive effect on problem-based learning (*β* = 0.30, *p* < 0.001), and problem-based learning has a positive effect on perceived employability (*β* = 0.27, *p* < 0.001). After future orientation and problem-based learning are both entered into Model 5, the results show that the relation between future orientation and perceived employability is reduced (*β* = 0.30, *p* < 0.001). Regarding the mediating effect, we tested the significance of the indirect effect using the bootstrapping technique (Shrout & Bolger, 2002). Specifically, as Table 4 shows, the bootstrapped confidence interval [95% CI: (0.04;0.12)] did not include zero. Therefore, the mediating effect was significant, supporting H2.

**Table 3:** Results of the mediation effects of problem-based learning

	Outcome variable: Problem-based learning		Outcome variable: Perceived employability		
	**Model 1**	**Model 2**	**Model 3**	**Model 4**	**Model 5**
**Control variables**					
Gender	0.07	0.03	-0.13^*^	-0.17^***^	-0.18^***^
Age	0.09	0.08	0.08	0.07	0.05
Internship experience	0.09	0.08	-0.01	-0.03	-0.05
**Independent variable**					
Future orientation		0.30^***^		0.38^***^	0.30^***^
**Mediator**					
Problem-based learning					0.27^***^
R^2^	0.02	0.11	0.02	0.17	0.23
△R^2^	0.02	0.09	0.02	0.15	0.06
F	2.58	11.23^***^	2.82^*^	18.31^***^	21.85^***^

*N* = 368; * *p* < 0.05, ***p < 0.001

**Table 4:** Direct and indirect effects of future orientation on perceived employability

Direct effect						
Effect 0.28^***^		SE 0.04	t 6.23		95%CI [0.26;0.44]	
**Indirect effect**						
Effect 0.07	Boot SE 0.02			Boot 95% CI [0.04;0.12]		

*N* = 368; *** *p* < 0.001

**Fig. 2:**
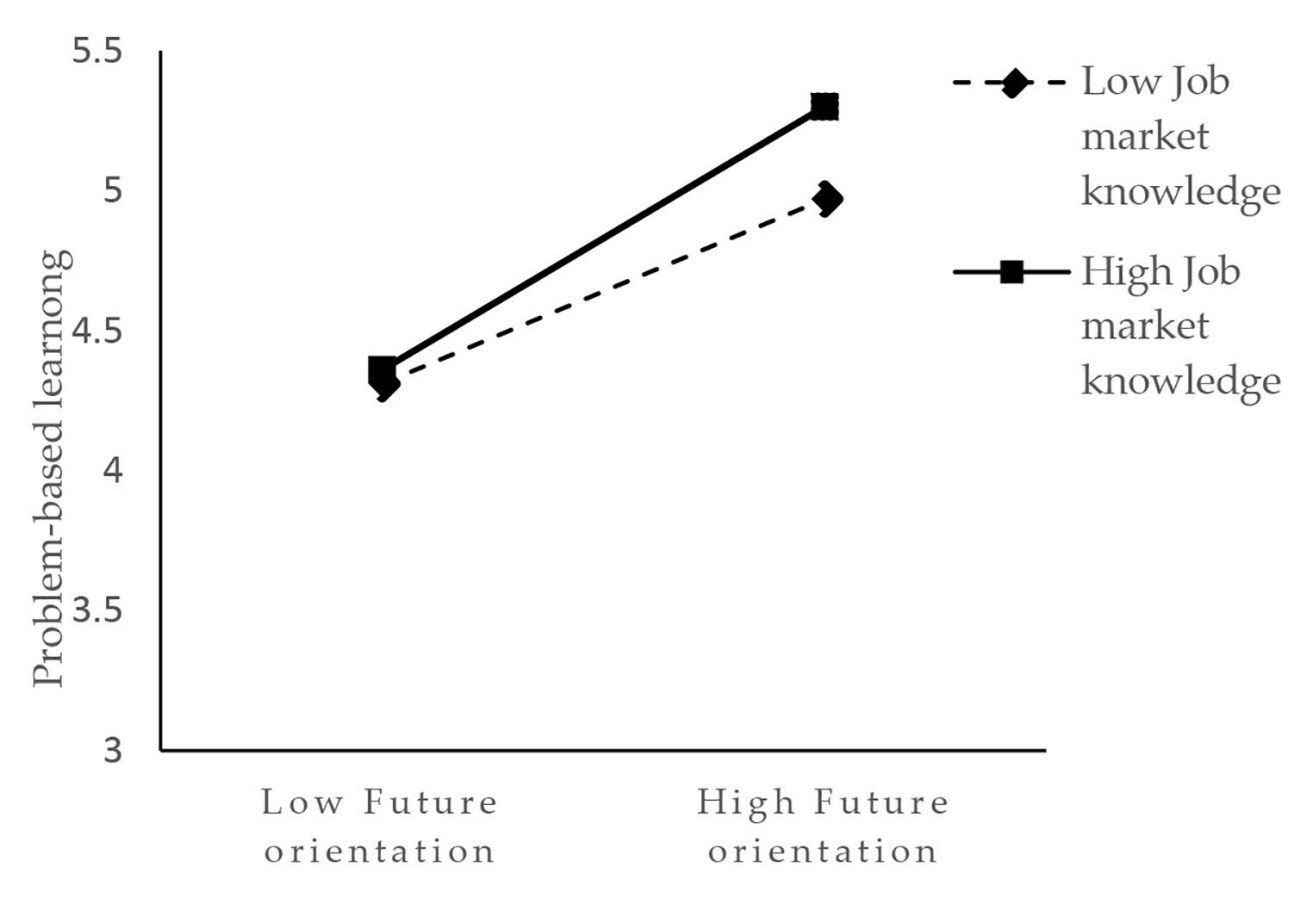
Low job market knowledge as a moderator in the relationship between future orientation and problem-based learning

**Fig. 3:**
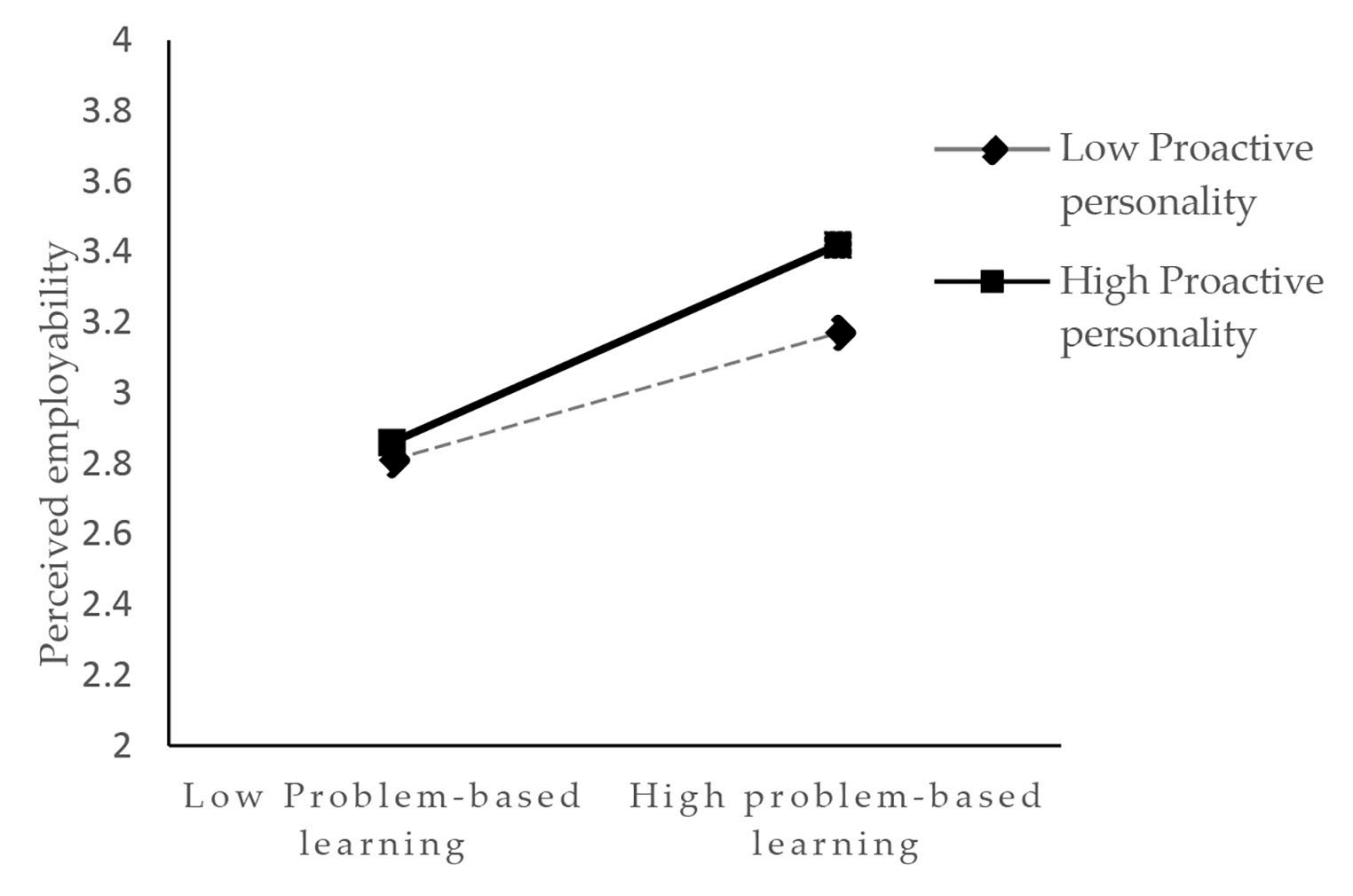
Proactive personality as a moderator in the relationship between problem-based learning and perceived employability

We used hierarchical regression analyses to test moderation effects. As shown in Table 5, the interaction term of “future orientation” × “job market knowledge” was positive and significant (*β* = 0.11, *p* < 0.05), which states that job market knowledge moderates the positive relationship between future orientation and problem-based learning. We also illustrated the pattern of the interaction effect in Fig. 2 to display the plot of the moderation effect, which showed that future orientation was significantly related to problem-based learning at both high levels (slope = 0.47, *p* < 0.001) and low levels (slope = 0.20, *p* < 0.05) of job market knowledge. Therefore, H3 is fully supported. Similarly, the interaction term of “problem-based learning” × “proactive personality” was positive and significant (*β* = 0.14, *p* < 0.01), thus supporting H4. Furthermore, Fig. 3 presents the results of simple slop test, which showed that problem-based learning was significantly related to perceived employability at both high levels (slope = 0.26, *p* < 0.001) and low levels (slope = 0.11, *p* < 0.01) of proactive personality.

**Table 5:** Results of the moderating effects of job market knowledge and proactive personality

		Outcome variable: Problem-based learning				Outcome variable: Perceived employability		
	**Model 1**		**Model 6**	**Model 7**	**Model 3**		**Model 8**	**Model 9**
**Control variables**								
Gender	0.07		0.05	0.05	-0.13		-0.16	-0.15
Age	0.09		0.05	0.05	0.08		0.03	0.02
Internship experience	0.09		0.07	0.06	-0.02		-0.06	-0.05
**Independent variable**								
Future orientation			0.23^***^	0.25^***^				
**Mediator**								
Problem-based learning							0.30^***^	0.31^***^
**Interactive effect**								
Job market knowledge			0.20^***^	0.20^***^				
Future orientation⊆Job market knowledge				0.11^*^				
Proactive personality							0.28***	0.28^***^
Problem-based learning⊆Proactive personality								0.14^**^
R^2^	0.02		0.14	0.15	0.02		0.23	0.25
△R^2^	0.02		0.12	0.01	0.02		0.20	0.02
F	2.58		11.99^***^	10.96^***^	2.82^*^		21.06^***^	19.58^***^

*N* = 368; ∗ *p* < 0.05, ∗∗ *p* < 0.01, ∗∗∗ *p* < 0.001

## Discussion

### Overview of findings

Exploring the relationship between students’ future orientation and perceived employability, we empirically found that students majoring in tourism and hospitality management with a future orientation tend to perceive a high level of employability in their future job market. We also observed a mediation effect, i.e., the relationship between future orientation and perceived employability is mediated by students’ problem-based learning. That is, when students are developing their future orientation, their problem-based learning increases, which in turn facilitates their perception of being employable in their future workplace.

Moreover, our results show that job market knowledge and proactive personality are moderators in the relationship between future orientation and perceived employability via problem-based learning. Specifically, job market knowledge positively moderates the relationship between future orientation and students’ problem-based learning; thus, when students have a higher level of job market knowledge, the effect of their future orientation on their problem-based learning becomes stronger. Meanwhile, proactive personality positively moderates the relationship between students’ problem-based learning and their perceived employability; hence, when students have more proactive personality, the effect of their problem-based learning on their perceived employability becomes stronger.

### Theoretical implications

The current research contributes to the literature in the following ways. First, as one of the first attempts to link students’ future orientation and their perceived employability in the tourism and hospitality field, we extend the literature on the association between students’ future orientation and their perceived employability (Tymon, 2013); that is, we enrich the existing knowledge on the predictors of students’ employability. Specifically, students with a more positive outlook of the future are more apt to design their future in terms of their career because they are more focused on their personal development and leisure time than on their future career choices (Ferrari et al., 2010; Ginevra et al., 2016). That is, they realize that their present constitutes the basis for the construction of their future (Ferrari et al., 2010; Laghi et al., 2009), rendering them more employable in their future job market. These findings are consistent with previous research that found that adolescents who tie the present to future career goals through future orientation will increase the likelihood of finding a “good enough job” (Ginevra et al., 2016). Thus, we address the need for a better understanding of how future orientation relates to career-related outcomes, such as employability (Tymon, 2013).

Second, our empirical findings concur with career construction theory; individuals who are more receptive to developing their careers are prone to engage in certain behaviors that support their career development (Savickas & Porfeli, 2012). These findings are consistent with previous studies, which found that individuals with positive personality traits (e.g., self-esteem and proactive personality) are more willing to actively explore their career development, and then tend to engage in behaviors that support their career development, such as career planning and career exploration(Cai et al., 2015; Valls et al., 2020). Moreover, by employing the dynamics of the vocational behavior paradigm within the framework of career construction theory (Cwr et al., 2019), we identify a behavioral mediator—problem-based learning—linking the relationship between students’ future orientation and their perceived employability. By opening the black box of the influence of future orientation on employability, we thus not only provide empirical findings that enrich the use of career construction theory in the employability literature (Forrier et al., 2015) but also underscore the empirical importance of the behavioral perspective for such research (Imam & Chambel, 2020).

Finally, by identifying the boundary conditions of job market knowledge and proactive personality, we address the question regarding when students’ future orientation contributes to their perceived employability by increasing their problem-based learning. Specifically, students who are highly equipped with job market knowledge are more likely to utilize their future orientation to learn how to address problems in their future workplace, acquiring the requisite knowledge or skills concerning their prospective job market and its employment trends to adequately prepare for potential problems in their future workplace. This suggests that students’ preparation for future work depends on personal-related career resources, such as job market knowledge, which aligns with career construction theory, where the personal factor is a key component (Savickas et al., 2005).

In line with theory that highlights the influence of personal characteristics (e.g., personality) on the development of career (Cwr et al., 2019) our results provide further evidence that proactive students are more apt to transfer their problem-based learning to a high level of employability in their future job market. This implies that desirable attributes can offer students more opportunities to take advantage of their strengths to develop their ability to obtain and retain a job, revealing the contingent role of proactive personality in employability from a theoretical perspective. These findings are consistent with previous research, which has shown that individuals with high initiative tend to create conditions for themselves to play to their strengths and pursue career goals set by themselves, which in turn has some positive effects on improving individual employability (Chughtai, 2019; Xia et al., 2020). Accordingly, our identification of two moderators with distinct paths increases the understanding of students’ employability by using personal-dependent variables to examine the unique relationship between future orientation and employability among students majoring in tourism and hospitality.

### Practical implications

Based on our findings, some practical implications can be identified to help students be more employable. First, universities should recognize the importance of developing students’ employability. For example, schools, especially truism and hospitality schools, should administer a range of programs to train students’ belief in their control over their future events. In addition, teachers in universities should act as facilitators by encouraging their students’ careers throughout their education, reinforcing the necessities of focusing on future jobs.

Our results show that problem-based learning is effective for helping students develop their perception of being employable. Therefore, teachers can design a problem-based learning scenario with an embedded problem that emerges through student brainstorming. Students are also encouraged to identify gaps in their knowledge, conduct research, and apply their learning to develop solutions and present their findings(Barrows, 1996).

Since job market knowledge can significantly strengthen the benefits of future orientation and problem-based learning, undergraduate education needs to be better aligned with the current job market in the training programs in the tourism and hospitality discipline. For instance, teachers should provide more knowledge on and help students practice the key skills and values that will be demanded by future employers through students’ coursework and extracurricular activities.

Finally, given the positive moderating role of proactive personality, universities should pay careful attention to the cultivation of students’ internal positive qualities, thereby fostering their proactive personality. For example, more opportunities should be provided to less proactive students to increase their participation in activities (e.g., answering questions and sharing ideas).

### Limitations and Future Research directions

There are, however, some limitations in the current study. The first limitation relates to sampling. That is, we collected data only from Chinese students in one Chinese university. To generalize our findings to the greatest extent, future research is encouraged to invite students from other universities in China and other cultural contexts. Moreover, although our research focuses on students majoring in tourism and hospitality management, our results can be applied to other disciplines. For example, in the educational field, students face similar problems of high risks in their workplace; therefore, future scholars could extend our current study by collecting data from students majoring in other fields.

Second, although we employed a time-lagged research design, causality may be a problem. Specifically, students’ future orientation and problem-based learning were both assessed at Time 1; thus, there is a potential influence of problem-based learning on students’ future orientation. That is, if a student is engaging in a problem-based learning process, he or she will obtain more knowledge for addressing problems in his or her future workplace (Cho et al., 2015); as a result, he or she is more likely to shift his or her focus on future work roles by developing a future orientation. Hence, students’ problem-based learning contributes to their future orientation. Future studies should thus establish the relationship between future orientation and perceived employability via problem-based learning. Specifically, future research can not only collect data at different stages (e.g., future orientation at time 1 and problem-based learning at time 2 with a six-month time interval) but also employ experimental methods to verify causal relationships.

The next limitation concerns CMB, as we collected data from only one source (i.e., students’ ratings). We therefore tested CMB and found that CMB is not a problem in our study. However, future studies are needed to exclude this problem through broadening others-ratings. For example, we recommend asking students’ coaches or teachers to evaluate the extent to which their students are employable in the job market.

Finally, we explored the boundary conditions of job market knowledge and proactive personality from the students’ personal perspectives. Future studies can thus account for some context-dependent variables to examine the indirect relationship between future orientation and perceived employability via problem-based learning. For instance, as most students lack work experience, their understanding of their future workplace is limited. Accordingly, teachers can act as an information source to help students gain more knowledge on how to prepare for their work in the future (e.g., being employable). This indicates that teacher-related factors might be a moderator that attenuates and/or strengthens the association between students’ personal factors and their employability.

## Conclusion

Bridging an existing research gap due to the limited research that has examined the association between students’ future orientation and their employability in the tourism and hospitality industries, in the current study, we hypothesize on and empirically examine why, how and when students’ future orientation can enhance their employability. Drawing on career construction theory, we propose that students majoring in tourism and hospitality management with a high level of future orientation generate a high level of perceived employability by increasing their problem-based learning, a process that is conditioned by job market knowledge and proactive personality. Conducting a time-lagged research design, we employed the validated measures of all the variables to increase the level of achievement achieved in the current study. We collected data from 368 Chinese students majoring in tourism and hospitality management at a Chinese university at two different time points with a one-month time interval. Our empirical findings indicate that problem-based learning acts as a mediator and that job market knowledge and proactive personality act as moderators in the relationship between students’ future orientation and their perceived employability.

## Data Availability

The data of this study are available from the corresponding author upon request.
